# Accuracy of death certification of dementia in population-based samples of older people: analysis over time

**DOI:** 10.1093/ageing/afy068

**Published:** 2018-04-28

**Authors:** Lu Gao, Rowan Calloway, Emily Zhao, Carol Brayne, Fiona E Matthews, Medical Research Council Cognitive Function and Ageing Collaboration

**Affiliations:** 1 MRC Biostatistics Unit, Cambridge Institute of Public Health, University of Cambridge, Cambridge, UK; 2 GP Vocational Training Scheme Hackney; 3 Department of Public Health and Primary Care, Cambridge Institute of Public Health, University of Cambridge, Cambridge, UK; 4 Faculty of Medicine, Institute of Health and Society, Newcastle University, Newcastle, UK

**Keywords:** death certification, dementia, population-based study, older people

## Abstract

**Background:**

death certification data are routinely collected in most developed countries. Coded causes of death are a readily accessible source and have the potential advantage of providing complete follow-up, but with limitations.

**Objective:**

to investigate the reliability of using death certificates for surveillance of dementia, the time trend of recording dementia on death certificates and predictive factors of recording of dementia.

**Subjects:**

individuals aged 65 and over in six areas across England and Wales were randomly selected for the Medical Research Council Cognitive Function and Ageing Study (CFAS) and CFAS II with mortality follow-up.

**Methods:**

prevalence of dementia recorded on death certificates were calculated by year. Reporting of dementia on death certificates compared with the study diagnosis of dementia, with sensitivity, specificity and Cohen’s κ were estimated. Multivariable logistic regression models explored the impact of potential factors on the reporting of dementia on the death certificate.

**Results:**

the overall unadjusted prevalence of dementia on death certificates rose from 5.3% to 25.9% over the last 26 years. Dementia reported on death certificates was poor with sensitivity 21.0% in earlier cohort CFAS, but it had increased to 45.2% in CFAS II. Dementia was more likely to be recorded on death certificates in individuals with severe dementia, or those living in an institution, yet less likely reported if individuals died in hospital.

**Conclusion:**

recording dementia on death certificate has improved significantly in the England and Wales. However, such information is still an underestimate and should be used alongside epidemiological estimations.

## Introduction

Mortality outcomes are considered to be reasonably robust to measurement error [1] and are useful for many cancer and vascular outcomes. As death certification data are routinely collected in most developed countries; coded causes of death made a readily accessible source, and the use of such data has the potential advantage of providing complete follow-up of those respondents who have given consent to track routine health records in prospective cohort studies, therefore enable many epidemiological investigation to be carried out in a much more cost-efficient manner.

Dementia is known to be associated with increased mortality, and the prevalence of dementia at death should therefore be higher than that in life. This has been empirically demonstrated [2, 3]. Dementia is, however known to be often omitted from death certificates as either the underlying cause or contributory cause [4] and is known to be more often recorded if it is severe, or death had occurred in a long-term care facility [4–6]. We performed a systematic literature search to identify publications in which any reporting dementia on death certificates was analysed using the population-based cohort. We found four new studies in addition to seven studies that were in an earlier systematic review [7]. The dementias on death certificates were under-reported in population-based cohorts, ranging from 7.2% to 44.4% [8]. A large number of studies of reporting dementia on death certification using patient cohorts were also found and have shown that, the sensitivity of death certificate recording of dementia for lifetime diagnosis of dementia varied enormously among studies across the world, from 8.7% to 93.2% (Supplementary Appendix Table A1, available at *Age and Ageing* online). The reliability of death certification of dementia can be assessed in well-designed longitudinal studies of patients with clinical diagnosis. Such studies are rare [5, 8–10] and needed for any interpretation of change over time.

Over the last 20 years, there have been dramatic changes in public awareness and policy regarding dementia. These have changed detection of dementia at primary care level and in acute hospital settings. In England and Wales, a registered medical practitioner who was in attendance during the deceased’s last illness, is required by legal obligation to certify the cause of death. One of the major changes to death certification recently is to avoid ‘old age’ alone, as clinically appropriate, record any medical conditions that may have contributed to the death. Doctors have been encouraged to note dementias on death certificates and dementia at the end of life has become a focus of interest. This has been highlighted particularly in the UK recently with publicity surrounding an Office of National Statistics (ONS) report [11] on increase of dementia on death registration, which was widely interpreted as supporting a parallel increase in dementia in the living.

The aim of this paper is therefore to evaluate the accuracy of death certification for dementia using two large population-based UK cohorts, to explore whether there have been changes over time on the dementia certification in relation to recent changes in practice, and to examine factors that affect the recording dementia on the death certificate.

## Methods

### Study design and population

Medical Research Council Cognitive Function and Ageing Study (CFAS) is a longitudinal population-based study started from 1989, with participants aged 65 years recruited in six areas across England and Wales [12, 13]. Briefly, a total of 26,699 people were randomly selected for the study, and 18,226 participants were interviewed at baseline with a schedule including socio-demographic items, general health and cognitive items. They were re-interviewed periodically either of the whole cohort or of sub-samples. The study closed in 2008 for active re-interview, though mortality information continues to be collected. A new cohort CFAS II started from 2008 using same study design and sampling method, 7,762 participants were recruited in the three of six CFAS original sites, and followed up with one wave of re-interviews 2 years later. Informant interviews were requested on all individuals who had a high probability of dementia or cognitive impairment from screen, all those who were unable to undertake or complete the interview. CFAS I and CFAS II interviewing has been given local and multi-centre ethical approval (CFAS I: MREC99/5/22, 05/MRE05/37; CFAS II: 07/MRE05/48).

All 26,699 participants who were eligible in CFAS and all 7,762 participants in CFAS II were flagged with the ONS for mortality and cause of death notification. Deaths up to the end of 2016 have been used in the current analyses.

All the deaths from CFAS and CFAS II were used for estimating the time trend of dementia certification, however for the investigation of agreement between the recording of dementia on the death certificates and the study diagnosis, only the individuals who had an interview in their last year of life were included in order that the diagnosis reflected as closely as possible their cognitive state in the run up to death, and to ensure sensitivity and specificity could be properly calculated [3]. For the investigation of factors associated with death certificate reporting of dementia, only those known dementia cases prior to death were used. In CFAS, follow-up interviews ceased in 2008, therefore those individuals who had an interview in their last year of life died before 2008, in CFAS II, all the deaths are after 2008, thus giving non-overlapping time frames for a comparison period from CFAS to CFAS II.

CFAS I and CFAS II interviewing has been given local and multi-centre ethical approval (CFAS I: MREC99/5/22, 05/MRE05/37; CFAS II: 07/MRE05/48).

### Definition of dementia

The study diagnosis of dementia uses the organic diagnosis syndrome of geriatric mental state-automated geriatric examination for computer assisted taxonomy [14], that has been validated against clinician’s diagnosis of dementia made according to Diagnostic and Statistical Manual of Mental Disorders III-R [15]. All individuals with organicity level 3 or above together with those with an interviewer’s recording of dementia were classified as with study diagnosis of dementia. The Blessed dementia scale [16] was used as an indicator of dementia severity.

During the study follow-up period, international classification of diseases (ICD)-10 was introduced in 2001 to replace ICD-9 at ONS. It was reported a difference between ICD-9 and ICD-10 for certain diseases including dementia [17]. Therefore, both ICD-9 (codes 290, 331.0, 298.9) and ICD-10 (F01, F03, G30, G318, F107) were used for identifying dementia on death certificates in order to accurately reflect the dementia diagnosis coded on the death certificates (see Supplementary Appendix for details, available at *Age and Ageing* online).

Dementia mentioned anywhere on a death certificate was used rather than only in underlying cause because dementia interacts with other conditions to predispose to early death. Mortality rates with dementia are much higher than mortality rates due to dementia. In addition, changes in the rules used by ONS to select the underlying cause of death from all of the conditions mentioned on the death certificate have a dramatic impact on mortality rates for particular conditions, including dementia [18].

### Statistical methods

Prevalence of dementia on death certificates was calculated from all deaths by year. Recordings of dementia on death certificates were compared with the study diagnosis of dementia, with sensitivity, specificity and Cohen’s κ were estimated separately in CFAS and CFAS II. Multivariable logistic regression models explored the impact of potential factors including age, place, and year of death, gender, living status (institution or in community), severity of dementia and geographical area on the reporting of dementia on death certificate.

## Results

**Figure 1.**
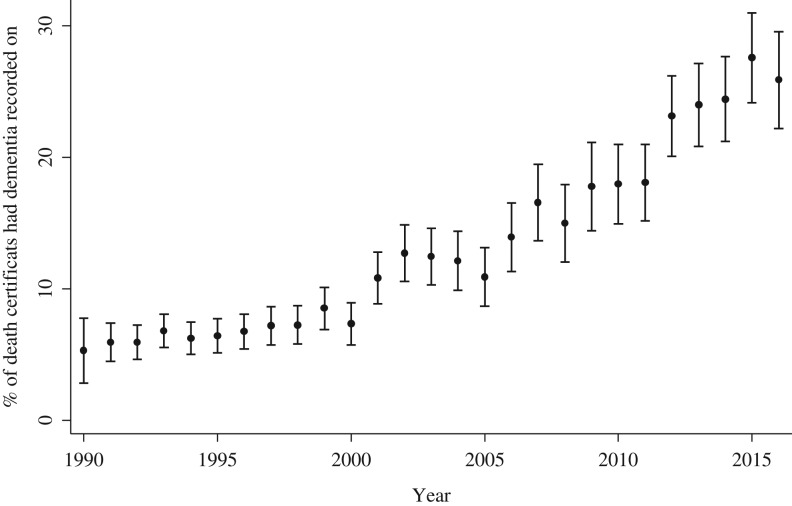
Prevalence dementia on death certificate over time. Bar is 95% CI.

In total, 24,506 participants (22,471 participants in CFAS I and 2,035 participants in CFAS II) had died by December 2016 for whom the death certificates are available. Dementia was mentioned on 2,847 (11.6%) death certificates (2,435 (10.8%) in CFAS I and 412 (20.3%) in CFAS II). Figure 1 shows the prevalence of dementia recorded on the death certificate from 1990 to 2016 has increased from 5.3% to 25.9% over a period of 26 years. The rise has been steady despite the ageing of the denominator up to 2008, then the resetting of the denominator with the new CFAS II study in 2008. The recording of dementia as underlying cause has also increased. As a proportion of all dementia mentioned on the death certificate, 38.0% had dementia as underlying cause before 2000, 49.7% between 2000 and 2009, and 71.3% after 2010. The majority of dementia is recorded as unspecified dementia (69.3%), followed by Alzheimer’s disease (21.6%), vascular dementia (8.6%) and other dementia (0.5%).

Of all the deceased participants, 3,236 have had an interview during their last year of life and known dementia status close to death (with or without dementia), those individuals were included for estimation of the sensitivity and specificity of recording dementia.

Table 1 reports the agreements between the dementia recorded on death certificate and the study diagnosis for CFAS and CFAS II separately as they represent two time periods. The estimate of sensitivity was 21.0% (95% CI: 18.4–23.8%) for CFAS, increasing to 45.2% (95% CI: 36.4–53.9%) for CFAS II. The estimates of specificity were both high. Cohen’s Kappa estimate of 0.26 (95% CI: 0.23–0.29) for CFAS indicates a fair agreement, with slightly improvement of 0.50 (95% CI: 0.41–0.59) for CFAS II rising to moderate agreement.

**Table 1.** Sensitivity and specificity of dementia reported on death certificates from participants who died within a year of last interview

On death certificate	CFAS study diagnosis		CFAS II study diagnosis	
	Not demented *N* (%)	Demented *N* (%)	Not demented *N* (%)	Demented *N* (%)
No dementia mentioned	1921 (99.5)	707 (79.0)	281 (97.9)	68 (54.8)
Dementia mentioned	9 (0.5)	188 (21.0)	6 (2.1)	56 (45.2)
	Estimate (95% CI)		Estimate (95% CI)	
Cohen’s Kappa (κ)	0.26 (0.23–0.29)		0.50 (0.41–0.59)	
Sensitivity	21.0 (18.4–23.8)		45.2 (36.4–53.9)	
Specificity	99.5 (99.1–99.8)		97.9 (96.3–99.6)	

Among the 15 death certificates that reported dementia for the participants who did not have study diagnosis of dementia before death, one had depression which might have masked the dementia; four had vascular dementia recorded, so the transition to dementia is likely to have occurred in the last year of life; and the others had already been experiencing some degree of cognitive impairment at the time of interview, thus it was possible that these participants had developed dementia after their last interview.

There were 1,580 deceased individuals with study diagnosis of dementia before death who also had information on the factors thought to influence reporting dementia on death certificate (subjects in Liverpool area were excluded due to missing Blessed score and information on residence). Table 2 summarises characteristics of these individuals by whether the dementia recorded or not on their death certificates, the odd ratios (OR) from multivariable logistic regression model. Those individuals living in an institution before death and individuals with severe or moderate dementia were more likely to have dementia recorded. Those individuals who died in hospital were less likely to have dementia recorded compared with those who died elsewhere. There was some centre variation though this was not significant, except Gwynedd in England and Wales. A sensitivity analysis including Liverpool showed further variation between centres (Supplementary Appendix Table A2, available at *Age and Ageing* online). The model shows that reporting dementia on death certificate over time has risen significantly compared with the reporting at the beginning of the study with OR of 3.3, 4.7 and 9.8 for the years of 2005–08, 2009–12 and 2013–16, respectively.

**Table 2.** Characteristics of individuals with study diagnosis of dementia and adjusted odd ratios from multiple logistic regression model of dementia recorded on death certificate

	Dementia recorded on death certificates (%)		Adjusted OR (95% CI)^a^
	No *N* = 1,051 (66.5)	Yes *N* = 529 (33.5)	
Centre^b^			
Cambridge	207 (62.0)	127 (38.0)	1
Gwynedd	184 (78.6)	50 (21.4)	0.6 (0.4–0.9)
Newcastle	227 (62.4)	137 (37.6)	0.9 (0.6–1.3)
Nottingham	238 (61.3)	150 (38.7)	1.0 (0.7–1.5)
Oxford	195 (75.0)	65 (25.0)	0.8 (0.6–1.2)
Sex			
Men	363 (70.1)	155 (29.9)	1
Women	688 (64.8)	374 (35.2)	1.1 (0.8–1.5)
Age-group at death			
≤74	43 (69.4)	19 (30.7)	1
75–84	301 (68.9)	136 (31.1)	1.0 (0.5–1.8)
85–94	548 (63.1)	321 (36.9)	1.1 (0.6–2.1)
≥95	159 (75.0)	53 (25.0)	0.5 (0.2–0.9)
Year of death			
1989–92	32 (82.1)	7 (18.0)	1
1993–96	333 (76.4)	103 (23.6)	1.5 (0.6–3.6)
1997–2000	275 (75.3)	90 (24.7)	1.6 (0.7–4.0)
2001–04	155 (69.8)	67 (30.2)	2.3 (0.9–5.7)
2005–08	81 (65.3)	43 (34.7)	3.3 (1.3–8.6)
2009–12	92 (52.6)	83 (47.4)	4.7 (1.9–11.8)
2013–16	83 (37.9)	136 (62.1)	9.8 (3.9–24.4)
Died in a hospital			
No	508 (58.6)	359 (41.4)	1
Yes	543 (76.2)	170 (23.8)	0.7 (0.5–0.9)
Lived in an institution			
No	462 (75.7)	148 (24.3)	1
Yes	585 (60.9)	375 (39.1)	1.8 (1.4–2.4)
Missing	4 (40.0)	6 (60.0)	
Blessed severity level			
Mild (0–5)	589 (75.3)	193 (24.7)	1
Moderate (6–11)	322 (64.7)	176 (35.3)	1.6 (1.2–2.1)
Severe (12–17)	140 (46.7)	160 (53.3)	3.6 (2.6–4.9)

^a^Adjusted for other factors.

^b^Centre Liverpool was not included.

## Discussion

This study based on unique population-based studies of dementia with mortality follow-up across two decades has provided the opportunity to examine changes in death certification practice for dementia in England and Wales. Three major findings emerged. Firstly, the overall estimate of prevalence of dementia on death certificates was 11.6%, with a dramatic 5-fold increase in recent years, but still significantly lowers than the prevalence of diagnosed dementia at death [3]. Secondly, the agreement between study diagnosis of dementia and recorded dementia on death certificates was poor in the earlier era becoming much less so more recently, however more than half of CFAS dementia were still not seen on death certificates. Thirdly, dementia recording on the death certificate is strongly associated with residential status leading up to death, severity of dementia, year of death and where death occurs.

The present analysis has some possible limitations. CFAS does not usually use clinical subtype, and comparisons by subtype of dementia cannot be investigated, as a previous study suggested the reporting of dementia was related to the clinical type of dementia [4, 6]. However, our own neuropathological studies indicate that in the oldest old, the presence of pure sub-types is unlikely and this use of such sub-types is open to question [19]. In addition, there is no confirmation of dementia using brain imaging either that may not manifest itself within the symptoms or to exclude symptoms that are not actually related to brain changes. The Blessed dementia scale was used as a measurement of dementia severity but was only available where informant interviews also available in smaller numbers. Even this reduced sample size is bigger than most other studies and has good statistical power. A sensitivity analysis excluding the Blessed and residence is provided in Supplementary Appendix Table A1, available at *Age and Ageing* online, with similar effects for all the other factors.

The increased prevalence of dementia on death certificates over time is unlikely to reflect a real change in dementia prevalence at death. There have been many factors that are likely to have influenced these changes. With greater awareness and changes for the medical profession encouraging the perception of dementia as a contributing cause of death. In our study, ICD-10 with potential diagnostic boundary shifts as well, seems to capture more dementia on death certificates, a finding also noted in previous publications that variation in coding could affect validity of dementia research [20].

Sensitivity has doubled over time: from 21.0% in CFAS where participants died before 2008, to 45.2% in CFAS II where deaths were after 2008, but more than half of those with a study diagnosis of dementia would be missed if death certificate dementia data alone were used to estimate prevalence. Those estimates cannot be relied on for planning health and social care for the people with dementia or even for those at the end of life as this would not accurately reflect need in the population.

Severity of dementia, living in an institution and place of death were found to predict the level of accuracy of recording dementia on death certificates, which reflect the facts that people with more severe dementia or who live in an institution, the dementia is more manifest. Conversely, dementia is more likely to be missed for those people who were admitted to a hospital for acute illness This is in agreement with earlier studies showing people with dementia developing bronchopneumonia and urinary tract infections, being hospitalised and dying where dementia is under-reported [21, 22]. Recent changes now require dementia screening on hospital admission so this may impact on these rates. Age effect on recording dementia was seen in the oldest age-band (95+) where less dementia was reported shows multiple co-morbidity may add challenges to diagnosis and reporting at very advanced ages.

The standard of death certification may be improved with changes of practice for completing cause of death, by recording all chronic conditions and mental illnesses that may have contributed to death. Providing data-linkage of information from patients’ medical history to assist in establishing underlying causes of death could give greater depth of knowledge. Understanding and awareness of importance of recording dementia on the death certificate by clinicians could increase dementia being recorded in these settings [23], and moving away from broad categories such as ‘old age’ or ‘senility’, thus providing reliable information in planning of hospital and community services.

## Conclusion

This analysis provides evidence that although under-reporting of dementia on the death certificate in England and Wales is declining dramatically but the use of death certificate information for dementia ascertainment is still likely to produce serious underestimates and is clearly unstable across time and is not recommended for such purpose. Therefore, it is unlikely that dementia certification on death certificate can be used as any marker of the true population level of dementia for many years.

**Key Points**

- The dementia recorded on death certificates has increased significantly raised from 5.3% to 25.9% over last two decades in England and Wales.
- Still less than half of those with dementia were recorded on death certificate.
- Dementia was more likely to be recorded on death certificates in individuals with severe dementia, or those living in an institution, yet less likely reported if individuals died in hospital.
- Death certification does not yet provide an alternative to more detailed population studies for estimation of dementia.
- The standard of death certification my be improved with changes of practice for completing causes of death.

## Supplementary Material

aa-17-0766-file003
