# Supplementary material for: Accuracy of death certification of dementia in population-based samples of older people: analysis over time
Source: Age Ageing. 2018 Apr 28;47(4):589–94. doi: 10.1093/ageing/afy068 (PMC6014308; doi:10.1093/ageing/afy068)
Supplement: aa-17-0766-file003 [file aa-17-0766-file003.docx]

**Appendix**

**Literature review**

The online search identified a total of 3254 articles

Figure 1: Literature review flow diagram

3156 articles not relevant based on title and abstract screen

Not included in review

5 Early results from included studies

16 No in life data

30 No Death certificate data

5 Other diseases not dementia

15 Mixed death and in life data

4 Review

4 No full text

**34 papers included in review**

3255 articles found in search

12 references from papers

112 papers for full text review

Table 1: Published studies reporting dementia on death certificates in samples of peoples of people with dementia

| Publication | Country | Year | Size | Sensitivity % | Specificity % | Note |  |
| --- | --- | --- | --- | --- | --- | --- | --- |
| **Population-based** |  |  |  |  |  |  |  |
| Ganguli [1] | US | 1987-2000 | 845 | 10.0 (6.9-14.0) | 99.6 (98.7-100) | MoVIES |  |
| Tschanz [2] | US | 1996-2001 | 1238 | 10.7 (7.4-14.8) | 98.3 (97.3-99.0) | Underlying cause |  |
| Ostbye [3] | US | 1995-2000 | 1410 | 22.0 (18.0-25.8) | 94.6 (93.1-95.8) |  |  |
| Nitrini [4] | Brazil | 1997-2000 | 40 | 17.5 (7.3-32.8) | NA | Includes senility |  |
| Romero [5] | Spain | 1994-2007 | 1976 | 20.8 (17.0-25.1) | 95.9 (94.8-96.9) | Underlying cause |  |
| Jin [6] | Sweden | 1987-2000 | 1200 | 28.2 (22.8-34.1) | 100 | Twin register, specificity as reported. 259 Dementia deaths |  |
| Morgan [7] | UK | 1985-1994 | 512 | 34.1 (20.5-49.9) | 95.7 (93.5-97.4) | Underlying cause |  |
| Ostbye [8] | Canada | 1991-1996 | 1539 | 37.6 (33.1-42.3) | 93.7 (92.0-95.0) | CSHA |  |
| Solomon [9] | Finland | 1998-2008 | 60 | 44.4 (13.7-78.8) | 100.0 (93.0-100) |  |  |
| **Other study types** |  |  |  |  |  |  |  |
| Dollear [10] | US | 1987-1990 | 37 | 8.7 (1.1-28.0) | 100.0 (76.8-100) | 23 stroke patients and 14 controls | |
| Macera [11] | US | 1988-1990 | 450 | 23.1 (19.3-27.3) | NA | Inpatient Alzheimer’s Register | |
| Bjertness[12] | Norway | 1990-1991 | 92 | 37.7 (26.3-50.2) | 100.0 (85.2-100) | Nursing home deaths | |
| Thomas [13] | UK | 1974-1994 | 348 | 49.7 (44.3-55.1) | NA | Vascular dementia – psychiatric hospital patients | |
| Perera [14] | UK | 2006-2013 | 7115 | 53.6 (52.5-54.8) | NA |  | |
| Wachterman [15] | US | 2003-2007 | 165 | 63.0 (55.2-70.4) | NA | Nursing home residents with end stage dementia | |
| Todd [16] | UK | 2002-2009 | 137 | 65.9 (54.9-75.8) | 96.2 (86.8-99.5) | Memory clinic / case control sample | |
| Newens [17] | UK | 1986-1992 | 257 | 65.0 (58.8-70.8) | NA | Pre-senile dementia register | |
| Martyn [18] | UK | 1981-1982 | 117 | 66.7 (57.4-75.1) | NA | Dementia clinic sample | |
| Raiford [19] | US | 1987-1993 | 200 | 67.5 (60.5-73.9) | NA | Probable/possible AD patients | |
| Burns [20] | UK | 1986-1990 | 84 | 70.2 (59.3-79.7) | NA | AD clinic patients | |
| Keene [21] | UK | 1988-2001 | 91 | 72.5 (62.1-81.4) | NA | Dementia patients at home with carers | |
| Kay [22] | UK | 1985-1998 | 192 | 72.4 (65.5-78.6) | NA | Pre-senile dementia register | |
| Olichney [23] | US | 1985-1992 | 97 | 76.3 (66.6-84.3) | NA | AD patients |  |
| Thomas [13] | UK | 1974-1994 | 398 | 90.4 (87.1-93.2) | NA | AD/Pre-senile AD – psychiatric hospital patients |  |
| Frecker [24] | Canada | 1987-1990 | 59 | 93.2 (83.5-98.1) | NA | Probable AD patients |  |
| **Excluded as provide biased sensitivity** | | | |  |  |  |  |
| Ives [25] | US | 1989-2004 | 3194 | 28.5 (23.8-33.6) | 99.4 (99.1-99.7) | Underlying cause  (Cause also used in study diagnosis not true sensitivity) |  |
| Zilkens [26] | Australia | 1990-2005 | 29884 | 55.9 (55.3-56.5) | NA | Hospital + DC ascertainment  (Cause also used in study diagnosis not true sensitivity) |  |
| Chamandy [27] | Canada | 1991-1996 | 2674 | 7.2 (5.4-9.2) | 99.5 (99.1-99.8) | CSHA Underlying cause |  |
| Stewart [28] | Canada | 1991-1996 | 66 | 33.0 (18.6-49.1) | 96.0 (80.4-99.9) |  |  |
| Ganguli [29] (CDR 0.5+)  (CDR 1+) | US | 1987-1996 | 527 | 23.8 (17.7-30.9)  33.3 (25.0-42.5) | 98.9 (97.1-99.7)  98.8 (97.2-99.6) |  |  |

**Dementia definition**

All individuals with organicity level 3 or above together with those with an interviewer’s recording of dementia at interview were classified as demented. In addition, for respondents who were with incomplete interviews or were not able to respond to the interview questions, interview data, interviewer rating and informant interview where present were reviewed in the same way for both studies (CB) and blind to death certification data.

During the study follow-up period, there has been a change of coding on death certificate at ONS. ICD-10 was introduced in 2001 to replace ICD-9. Cano-Serral and colleagues reported a difference between ICD-9 and ICD-10 for certain diseases including senile dementia. Therefore both ICD-9 and ICD-10 were used for identifying dementia on death certificates in order to accurately reflect the dementia diagnosis coded on the death certificates. When using ICD-9 coding, dementia was considered recorded if any of the code 290 (senile and pre-senile organic psychotic conditions) or 331.0 (Alzheimer’s disease) appeared anywhere on the death certificate. Dementia was also assumed for code 298.9 (unspecified non-organic psychosis) since we found that text entries for this coding included the word “dementia”; moreover, it is already known that 298.9 has been used by neurologists and at ONS to code dementia. For ICD-10 coding, dementia was assumed if F01 (Vascular Dementia), F03 (Dementia) or G30 (Alzheimer’s Disease) appeared anywhere on the death certificates, in addition, G318 (Lewy Bodies Dementia) and F107 (alcohol related dementia) were also coded as dementia in this analysis.

The Blessed dementia scale (range 0-17) was used in informant interviews as a measurement of activities of daily living and thus as an indicator of dementia severity. Participants were categorized into three groups based on their Blessed score as mildly (0 – 5), moderately (5.5 - 11) or severely (11.5 – 17) demented for this analysis.

Table 2: Characteristics of individuals with study diagnosis of dementia (all centres), and adjusted odd ratios from multiple logistic regression model of dementia recorded on death certificate

|  | Dementia recorded  on death certificates (%) | | Adjusted*  OR (95 % CI) | |
| --- | --- | --- | --- | --- |
|  | Yes  N=992 (31.8) | No  N=2,129 (68.2) |  |  |
| Centre |  |  |  |  |
| Cambridge | 165 (35.1) | 305 (64.9) | 1.0 |  |
| Gwynedd | 71 (21.3) | 263 (78.7) | 0.6 | (0.4 - 0.9) |
| Newcastle | 171 (36.2) | 302 (63.8) | 1.0 | (0.7 – 1.3) |
| Nottingham | 183 (36.0) | 325 (64.0) | 1.0 | (0.8 – 1.3) |
| Oxford | 93 (25.5) | 272 (74.5) | 0.8 | (0.6 – 1.0) |
| Liverpool | 279 (31.4) | 651 (68.6) | 1.2 | (0.9 – 1.5) |
| Sex |  |  |  |  |
| Men | 328 (30.9) | 744 (69.1) | 1.0 |  |
| Women | 634 (32.2) | 1,374 (67.8) | 1.1 | (0.9 – 1.3) |
| Age-group at death |  |  |  |  |
| <=74 | 31 (29.4) | 79 (70.6) | 1.0 |  |
| 75-84 | 252 (32.6) | 536 (67.4) | 1.0 | (0.6 – 1.5) |
| 85-94 | 579 (34.1) | 1,125 (65.9) | 0.9 | (0.6 – 1.4) |
| >=95 | 101 (22.1) | 375 (77.9) | 0.4 | (0.3 – 0.7) |
| Year of death |  |  |  |  |
| 1989 - 2000 | 500 (24.20) | 1,566 (75.8) | 1.0 |  |
| 2001 - 2016 | 462 (45.6) | 552 (54.4) | 2.9 | (2.4 – 3.5) |
| Died in a hospital |  |  |  |  |
| No | 614 (35.2) | 1129 (64.8) | 1.0 |  |
| Yes | 348 (26.0) | 1. 4.0) | 0.7 | (0.6 – 0.8) |

*Adjusted for other factors

Reference List

1 Ganguli M, Dodge HH, Shen C, *et al.* Alzheimer Disease and Mortality. *Arch Neurol* 2005;**62**:779. doi:10.1001/archneur.62.5.779

2 Tschanz JT, Corcoran C, Skoog I, *et al.* Dementia: the leading predictor of death in a defined elderly population: the Cache County Study. *Neurology* 2004;**62**:1156–62. doi:10.1212/01.WNL.0000118210.12660.C2

3 Østbye T, Taylor DH, Clipp EC, *et al.* Identification of dementia: Agreement among national survey data, medicare claims, and death certificates. *Health Serv Res* 2008;**43**:313–26. doi:10.1111/j.1475-6773.2007.00748.x

4 Nitrini R, Caramelli P, Herrera E, *et al.* Mortality from dementia in a community-dwelling Brazilian population. *Int J Geriatr Psychiatry* 2005;**20**:247–53. doi:10.1002/gps.1274

5 Romero JP, Benito-León J, Mitchell AJ, *et al.* Under reporting of dementia deaths on death certificates using data from a population-based study (NEDICES). *J Alzheimer’s Dis* 2014;**39**:741–8. doi:10.3233/JAD-132765

6 Jin Y-P, Gatz M, Johansson B, *et al.* Sensitivity and specificity of dementia coding in two Swedish disease registries. *Neurology* 2004;**63**:739–41.http://www.ncbi.nlm.nih.gov/pubmed/15326258 (accessed 24 Aug2017).

7 Morgan K, Clarke D. To what extent is dementia underreported on British death certificates? *Int J Geriatr Psychiatry* 1995;**10**:987–90. doi:10.1002/gps.930101112

8 Ostbye T, Hill G, Steenhuis R. Mortality in elderly Canadians with and without dementia: a 5-year follow-up. *Neurology* 1999;**53**:521–6.http://www.ncbi.nlm.nih.gov/pubmed/10449114 (accessed 24 Aug2017).

9 Solomon A, Ngandu T, Soininen H, *et al.* Validity of dementia and Alzheimer’s disease diagnoses in Finnish national registers. *Alzheimer’s Dement* 2014;**10**:303–9. doi:10.1016/j.jalz.2013.03.004

10 Dollear W, Gorelick PB, Harris Y, *et al.* Vascular dementia: a clinical and death certificate study. *Neuroepidemiology* 1992;**11**:53–8.http://www.ncbi.nlm.nih.gov/pubmed/1495574 (accessed 24 Aug2017).

11 Macera CA, Sun RK, Yeager KK, *et al.* Sensitivity and specificity of death certificate diagnoses for dementing illnesses, 1988-1990. *J Am Geriatr Soc* 1992;**40**:479–81.http://www.ncbi.nlm.nih.gov/pubmed/1634701 (accessed 24 Aug2017).

12 Bjertness E, Torvik A, Ince PG, *et al.* Validation of Norwegian death certificates on dementia in residents of nursing homes. *Epidemiology* 1998;**9**:584–6.http://www.ncbi.nlm.nih.gov/pubmed/9730045 (accessed 24 Aug2017).

13 Thomas BM, Starr JM, Whalley LJ. Death certification in treated cases of presenile Alzheimer’s disease and vascular dementia in Scotland. *Age Ageing* 1997;**26**:401–6.http://www.ncbi.nlm.nih.gov/pubmed/9351485 (accessed 24 Aug2017).

14 Perera G, Stewart R, Higginson IJ, *et al.* Reporting of clinically diagnosed dementia on death certificates: Retrospective cohort study. *Age Ageing* 2016;**45**:667–72. doi:10.1093/ageing/afw077

15 Wachterman M, Kiely DK, Mitchell SL. Reporting dementia on the death certificates of nursing home residents dying with end-stage dementia. *JAMA* 2008;**300**:2608–10. doi:10.1001/jama.2008.768

16 Todd S, Barr S, Passmore AP. Cause of death in Alzheimer’s disease: a cohort study. *QJM* 2013;**106**:747–53. doi:10.1093/qjmed/hct103

17 Newens a J, Forster DP, Kay DW. Death certification after a diagnosis of presenile dementia. *J Epidemiol Community Health* 1993;**47**:293–7. doi:10.1136/jech.47.4.293

18 Martyn CN, Pippard EC. Usefulness of mortality data in determining the geography and time trends of dementia. *J Epidemiol Community Health* 1988;**42**:134–7. doi:10.1136/jech.42.2.134

19 Ramadan NM. Postmeningitic cluster headache CERAD part VII : Accuracy of reporting dementia on death certificates of patients with Alzheimer ’ s disease Intrathecal baclofen treatment for stiff-man syndrome : Pump failure may be fatal. 1994.

20 Burns A, Luthert P, Levy R, *et al.* Accuracy of clinical diagnosis of Alzheimer’s disease. *BMJ* 1990;**301**:1026.http://www.ncbi.nlm.nih.gov/pubmed/2249050 (accessed 24 Aug2017).

21 Keene J, Hope T, Fairburn CG, *et al.* Death and dementia. *Int J Geriatr Psychiatry* 2001;**16**:969–74.http://www.ncbi.nlm.nih.gov/pubmed/11607941 (accessed 24 Aug2017).

22 Kay DWK, Forster DP, Newens a. J. Long-term survival, place of death, and death certification in clinically diagnosed pre-senile dementia in northern England: Follow-up after 8-12 years. *Br J Psychiatry* 2000;**177**:156–62. doi:10.1192/bjp.177.2.156

23 Olichney JM, Hofstetter CR, Galasko D, *et al.* Death certificate reporting of dementia and mortality in an Alzheimer’s disease research center cohort. *J Am Geriatr Soc* 1995;**43**:890–3.http://www.ncbi.nlm.nih.gov/pubmed/7636097 (accessed 24 Aug2017).

24 Frecker MF. Dementia in Newfoundland: identification of a geographical isolate? *J Epidemiol Community Health* 1991;**45**:307–11. doi:10.1136/jech.45.4.307

25 Ives DG, Samuel P, Psaty BM, *et al.* Agreement between nosologist and cardiovascular health study review of deaths: Implications of coding differences. *J Am Geriatr Soc* 2009;**57**:133–9. doi:10.1111/j.1532-5415.2008.02056.x

26 Zilkens RR, Spilsbury K, Bruce DG, *et al.* Linkage of hospital and death records increased identification of dementia cases and death rate estimates. *Neuroepidemiology* 2008;**32**:61–9. doi:10.1159/000170908

27 Chamandy N, Wolfson C. Underlying cause of death in demented and non-demented elderly Canadians. *Neuroepidemiology* 2005;**25**:75–84. doi:10.1159/000086287

28 Stewart M, McDowell I, Hill G, *et al.* Estimating antemortem cognitive status of deceased subjects in a longitudinal study of dementia. *Int Psychogeriatr* 2001;**13 Supp 1**:99–106. doi:10.1017/S1041610202008037

29 Ganguli M, Rodriguez EG. Reporting of dementia on death certificates: a community study. *J Am Geriatr Soc* 1999;**47**:842–9.http://www.ncbi.nlm.nih.gov/pubmed/10404929 (accessed 24 Aug2017).
